# Significance of High Levels of Endogenous Melatonin in Mammalian Cerebrospinal Fluid and in the Central Nervous System

**DOI:** 10.2174/157015910792246182

**Published:** 2010-09

**Authors:** Dun-Xian Tan, Lucien C Manchester, Emilio Sanchez-Barcelo, Maria D Mediavilla, Russel J Reiter

**Affiliations:** Department of Cellular & Structural Biology, University of Texas, Health Science Center, at San Antonio, 7703 Floyd Curl, San Antonio, TX, 78229, USA

**Keywords:** Melatonin, pineal gland, CNS, CSF, oxidative stress, neurodegenerative disease.

## Abstract

Levels of melatonin in mammalian circulation are well documented; however, its levels in tissues and other body fluids are yet only poorly established. It is obvious that melatonin concentrations in cerebrospinal fluid (CSF) of mammals including humans are substantially higher than those in the peripheral circulation. Evidence indicates that melatonin produced in pineal gland is directly released into third ventricle *via *the pineal recess. In addition, brain tissue is equipped with the synthetic machinery for melatonin production and the astrocytes and glial cells have been proven to produce melatonin. These two sources of melatonin may be responsible for its high levels in CNS. The physiological significance of the high levels of melatonin in CNS presumably is to protect neurons and glia from oxidative stress. Melatonin as a potent antioxidant has been reported to be a neuroprotector in animals and in clinical studies. It seems that long term melatonin administration which elevates CSF melatonin concentrations will retard the progression of neurodegenerative disorders, for example, Alzheimer disease.

## INTRODUCTION

Melatonin is a secretory product of pineal gland in mammals and it is synthesized by pinealocytes beginning with the essential amino acid, tryptophan. Its isolation and structural identification by Lerner *et al.* [29] have been great contributions to all fields of biology and medicine. Since its discovery, a large variety of physiological functions of melatonin have been uncovered. These include melatonin synchronization of circadian and seasonal rhythms, regulation of the reproductive activity in photoperiodic species [48], defending against oxidative stress [69], balancing organismal energy metabolism [17, 58, 67] and retarding the aging process [9, 64, 67].

In addition to the pineal gland, melatonin is found in a large number of extrapineal tissues and organs which also have the capacity to biosynthesize the indoleamine. Cells and organs that produce melatonin include astrocytes, glial cells, lymphocytes, retinal cells, gut, testes, ovary, placenta, skin, etc [69]. Among them, the gut and skin are the largest organs to produce melatonin. The extrapineal derived-melatonin, however, seems to play a little role in the classic blood  melatonin circadian rhythm due to the fact that pinealectomy diminishes this rhythm [46]. It has been speculated that melatonin of extrapineal origin is consumed by the tissues or organs where melatonin is produced as a defense against the oxidative stress [69]. 

Recent studies have also documented that a variety of food stuffs, e.g., vegetables, cereals, fruits, nuts, seeds, grapes, red wine and beer contain considerable amounts of melatonin [25, 26, 37, 38, 45]. Food consumption in some cases alters the blood melatonin levels [22]. There are no data indicating that food-derived melatonin or postprandially-absorbed melatonin significantly alter circadian or seasonal physiological rhythms. 

In recent decades, it was commonly accepted that the physiological levels of melatonin in the blood represent the levels throughout the body. The blood values range from several pg/ml during the day to more than 50 pg/ml at its nighttime peak. These levels, however, do not reflect the real concentrations of melatonin in tissues or in other body fluids. These values significantly underestimate the levels of melatonin *in vivo*. For example, the gut produces several hundred-fold more melatonin than the pineal gland generates [24]; this melatonin in retained in gut tissue where levels are higher than in the blood. In bone marrow of rats much higher levels of melatonin also have been found [68]; this is also true for bile of the human and other mammals [65], ovarian follicular [8] and amniotic fluid of women [42] and cerebrospinal fluid of sheep [62, 71]. The real melatonin levels in different tissues or body compartments remain virtually unknown. In the current review, what is known regarding the levels of melatonin in central nervous system (CNS) is discussed and the physiological significance of melatonin in the mammalian CNS is also addressed.

## ORIGIN OF MELATONIN IN CNS

While the pathway for melatonin synthesis is well documented, there is some controversy concerning the rate limiting enzyme in its production. The essential enzymes in the melatonin pathway are arylalkylamine N-acetyltrans-ferase (AANAT) and hydroxyindole O-methyltransferase (HIOMT). The AANAT is often portrayed as the rate limiting enzyme but this may not be the case under all circumstances [32] and HIOMT also plays a crucial role to control melatonin synthesis by converting N-acetylserotonin to melatonin [13, 32, 52]. Historically, melatonin originating from the pineal gland was believed to be the sole source of melatonin in the blood and CNS and the pineal gland secretion was responsible for melatonin fluctuations in the CSF of mammals. Recent studies indicate that pineal-derived melatonin may not be the only resource of melatonin in CNS. The mRNAs of AANAT and HIOMT have been indentified in the brain tissue of rats [63]. This indicates that that brain may possess the machinery for melatonin synthesis. Whether the neurons in fact synthesize melatonin is an open question. The astrocytes of rats and the human glioma C6 cellline, however, have been found to produce melatonin under *in vitro* conditions [33].

On other hand, extremely high levels of N^1^-acetyl-N^2^-formyl-5-methoxykynuramine (AFMK), a unique melatonin metabolite, have been measured in the CSF of patients with meningitis [59]. The levels of AFMK in those patients are several orders of magnitude higher than the melatonin levels in the CSF of normal subjects. AFMK was actually identified in brain of rats decades ago [23] and might be the major metabolite of melatonin in neurons or in other systems [69]. Due to the fact that one melatonin molecule only forms one AFMK molecule, it appears that the levels of AFMK mentioned above exceed the productive capacity of pineal-derived melatonin. Considering this, it is likely that both pineal and extrapineal, i.e., brain tissue, melatonin contribute to the level of this indolamine in the CSF.`

As an effective antioxidant and neuroprotector [11, 12, 39] the synthesis of melatonin may be inducible as a result of oxidative stress or other stresses. The phenomenon of stress-induced melatonin production has been observed in plants [2], pancreas of rats [28] and in human CSF after traumatic brain injury [55]. Stress-induced melatonin production may be an explanation for the high levels of AFMK in the CSF of patients with meningitis. 

## PINEAL GLAND AND MELATONIN IN THE CSF

**Fig. (1):**
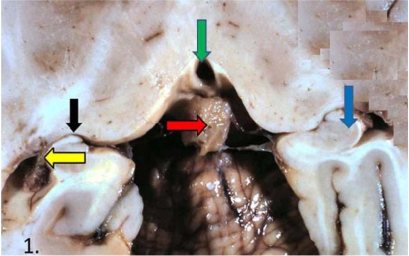
Human pineal gland in related to its surrounding structures. Horizontal brain section. Red arrow – pineal gland with surrounding basal cistern. Green arrow – third ventricle. Black arrow –the choroid fissure. Yellow arrow – choroid plexus of the inferior horn of the lateral ventricle. Blue arrow – hippocampus. Modified from Maurizi [41].

The human pineal gland is located near the center of the brain and is surrounded with basal cistern, ventricles, choroid fissure and choroid plexus (Fig. **1**).

The relationship of pineal gland with surrounding structures is an interesting issue and has been recently re-examined. Roughly four decades ago, Sheridan *et al.* [56, 57] unambiguously identified the pineal recess of the “deep pineal gland” and noted that it directly contacts the third ventricle in the hamster. At that time, however, the physiological significance of this recess, which is an evagination of the third ventricle was unknown. It was believed that the CSF melatonin was derived exclusively from the peripheral circulation, i.e., pineal melatonin is secreted into the Galen vein, which drains into the sagittal sinus, jugular vein and then into the general circulation. Thereafter, circulating melatonin was finally transported back to the CSF *via *the cerebral arteries to enter the ventricular system after its release by the choroid plexus. Thus, melatonin concentrations in CSF were expected to be similar to those in the peripheral blood. This concept has been challenged by a discovery that the melatonin in CSF exhibits a concentration gradient in sheep [71]. The highest concentration is measured in the third ventricle near the pineal recess in the sheep ventricular system; thereafter, the concentrations of melatonin gradually decrease in CSF collected from the center of the third ventricle, aqueduct, fourth ventricle and lumbar subarachnoid space. Likewise, melatonin concentrations are lower in the lateral ventricles than in the third ventricle. A similar melatonin concentration gradient in the CSF of humans has also been observed [35]. 

It is well known that the direction of flow of CSF is from the lateral ventricles through the interventricular foramina into the third ventricle, to the aqueduct and to the fourth ventricle. It is assumed that the high level of melatonin in the third ventricle near the pineal recess is gradually diluted by the CSF flow. Melatonin in the lateral ventricles is probably derived from the melatonin that diffuses against the current from the third ventricle and may also be released from the choroid plexi which are prominent in these ventricles. When the pineal recess was surgically sealed, the high concentration of melatonin in the third ventricle was markedly reduced [71]. These are consistent with the idea that a major portion of CSF melatonin is directly released into the third ventricle from the pineal gland *via *the pineal recess rather than being derived from the peripheral circulation. 

Since the anatomy of the pineal gland and its surrounding structures is similar in sheep and in humans, it is reasonable to assume that the majority of melatonin in the CSF of humans also comes directly from the pineal gland. The anatomic evidence of a direct connection of pineal gland with CSF in humans has recently been highlighted by Maurizi [41]. This investigator argues that the shunting of pineal derived-melatonin directly into the ventricular system should be taken seriously.

A clinical study has recently reported that melatonin concentrations in third ventricle of patients with movement disorders are significantly higher than in lateral ventricles and in blood. The authors argue that pineal melatonin is likely directly released into the third ventricle in humans [30]. While much of the melatonin may be of pineal origin, other CNS sources of CSF melatonin should not be ignored. These sources include melatonin from the peripheral circulation and melatonin synthesized by brain tissue, especially, under the stressful conditions such as in a brain inflammatory response [59] or in brain traumatic injury [55].

## LEVELS OF MELATONIN IN CSF

Measurement of the levels of melatonin in ventricular CSF is complicated by the fact that third ventricular CSF is difficult to collect in humans. Extracting CSF from the ventricular system is obviously not a routine procedure and when it is done, it is usually after serious injury to the brain. Several studies have documented different concentrations of CSF melatonin in rats, in sheep and in humans with a damaged or diseased brain. Also, marked differences in CSF concentrations in different species or within individuals of the same species should also be expected. These differences may partially a result of the methodologies which are used to detect melatonin, partially result from the location where the CSF is collected and also the time when the CSF is extracted.

As to the methodologies used to measure melatonin, the high performance liquid chromatograph ( HPLC) plus mass spectrum (MS) technologies are more specific and accurate than radioimmunoassay or ELISA. Complicating the measurement is that two forms of melatonin, i.e., free and protein- bound melatonin are present in the CSF. The amounts of bound melatonin are several-fold greater than the free melatonin in CSF [53]. Route HPLC methods only detect free melatonin and, thus, the melatonin levels in CSF are usually significantly underestimated. 

The site of CSF collection is also important when estimating melatonin concentrations. As mentioned previously, there is a melatonin concentration gradient in CSF throughout the third ventricle. When CSF is obtained *via *the lumbar puncture, which is often the case, melatonin levels would be expected to be significantly lower than CSF collected from the ventricles of the brain. 

The time of CSF collection is another important factor impacting CSF melatonin levels. Melatonin levels in CSF, as in the blood, exhibit a circadian rhythm with a peak at night and basal levels during the day [18]. For most human studies, the CSF is collected during the daytime and invariably the nighttime rise is missed. The highest nighttime melatonin concentration in CSF has been reported in sheep; in this case, the levels were 19,934 ± 6,388 pg/ml [71]. These levels are several hundred-fold higher than the melatonin concentrations measured in simultaneously-collected blood samples. For a comparison of melatonin concentrations in human CSF, the results of several studies are summarized in Table **1**.

As indicated in this table, the majority of these studies are performed during the day or post mortem. Even in the single case where CSF *via *lumbar punctured was collected at night, the patient was in light at the time. Under these conditions, melatonin levels are either at their basal values or in the process of degradation. In addition, most of these studies only measured the free melatonin present in the CSF. Based on the findings reported by Rizzo *et al.* [53], the free melatonin only comprised one fourth of the total melatonin in the CSF. Thus, it seems that melatonin levels in CSF are in fact much higher than the values currently published. It is also obvious that the melatonin levels in CSF far exceed these measured in the serum at the same time. 

## SIGNIFICANCE OF HIGH LEVELS OF MELATONIN IN CSF

Melatonin is a pleiotropic molecule that plays several important roles in CNS including circadian rhythm regulation [20], sleep promotion and blood pressure modification [60]. These actions are probably mediated by the membrane melatonin receptors which are located in a variety of cells in the CNS. Several excellent publications have reviewed the functions of melatonin in the brain and the interested readers should consult these articles [10, 19, 27].

In the current review, we address the neuroprotective effects of melatonin. These actions of melatonin are in part based on the discovery that melatonin is a potent endogenous free radical scavenger and antioxidant [66]. It is well known that CNS is an ready target for oxidative stress since brain consumes large amounts of oxygen; therefore, it produces more reactive oxygen species (ROS) than other organs and tissues. If these ROS are not scavenged or detoxified by any of a number of antioxidants, neuronal cells are injured by a process which is referred to as oxidative stress or nitrosative stress. Many neurodegenerative disease including Alzheimer disease, Parkinson disease and amyotrophic lateral sclerosis (ALS) are at least in part related to neuronal oxidative damage and cell loss [43, 47]. Melatonin is one of the best endogenously-occurring molecules that protect the brain from such damage. Melatonin not only has the capacity to scavenge a variety of ROS and reactive nitrogen species (RNS) including hydroxyl radical (HO^**.**^), superoxide anion radical (O_2_^**.-**^) hydrogen peroxide (H_2_O_2_), nitric oxide (NO^**.**^) and peroxynitrite anion (ONOO^**-**^) [70], but also it up-regulates gene expression and stimulates the activities of several antioxidant enzymes, including glutathione peroxidase, superoxide dismutase and catalase [49, 50]. In addition, melatonin acts at the level of electron transport chain of mitochondria to inhibit ROS formation [1]; this is referred as the free radical avoidance effect of melatonin [21].

The potential associations of physiological levels of melatonin in CSF and neurodegenerative disease, e.g, Alzheimer disease, have been reported. Several clinical investigations have shown that melatonin concentrations in CSF of Alzheimer patients are several-fold lower than those in age-matched non-Alzheimer control subjects [31, 61, 72]. CSF melatonin levels in patients with Alzheimer disease are negatively correlated with disease status, i.e, the more severe the disease, the less melatonin is present in CSF [72]. Currently, it remains unknown whether the low levels of melatonin in the CSF of Alzheimer patients is the result of reduced melatonin production in these patients or elevated melatonin metabolism related to the disease status since increased oxidative stress consumes more melatonin. 

Several small scale and non-double-blinded clinical trials have tested the treatment effect of melatonin as a powerful antioxidant in Alzheimer disease. Some promising results have been obtained from these clinical studies. These studies show that the oral administration of 6-9 mg melatonin at bed time significantly retards the progression of the disease, reduces symptoms such as sundowning and modifies the sleep pattern of the patients [3, 6, 7, 16, 36]. These preliminary observations require confirmation in large scale and well-controlled clinical trials. Nevertheless, these preliminary and promising results warrant further research in terms of the use of melatonin to treat Alzheimer disease and other neurodegenerative disorders.

In addition to the human studies, the protective effects of melatonin on brain damage caused by a variety of processes have been intensively investigated in animal studies. Melatonin administration or pineal grafts into the brain significantly reduced the infarct volume in the rat brain induced by the middle cerebral artery ischemia/reperfusion [5, 14]. In the transgenic animal models of Alzheimer disease, long term melatonin supplementation not only protected against cognitive deficits and indices of neurodegeneration but also prolong the survived period [15, 40, 44]. The neuroprotective effects of melatonin are mainly attributed by its powerful antioxidant capacity [51]. 

An obvious advantage of melatonin in neurodegenerative diseases is its ready permeability into the CNS. Melatonin as a lipophilic and hydrophilic molecule [4] passes the blood-brain barrier with ease. A clinical study shows that 10 min after oral melatonin intake, a melatonin peak is observed in the CSF and a relatively high level of melatonin is maintained for several hours [18]. The elevated melatonin level in CSF is beneficial to the brain tissue around the ventricles in terms of oxidative stress, especially in neurodegenerative conditions such as Alzheimer disease where the level of oxidative damage is increased. Based on anatomical relationships of the pineal gland with the ventricular system and the CSF, Maurizi [41] concluded that “ the elevated levels of melatonin in the CSF would be translocated into neurons, protecting these cells from oxygen free radical damage”; he also reminded the reader that “a prudent shopper can buy a year’s supply of supplemental melatonin tablets which provide a dose of 9 mg daily, which seems to slow the progression of the Alzheimer disease, for less than $25”. 

## CONCLUSION REMARKS

Evaluation of the melatonin levels in tissues and other body fluids based on the blood melatonin concentrations appears to be inadequate since the distribution of melatonin in the body is not homogenous. Several studies have shown that levels of melatonin in CSF are much higher than those in the blood. Evidence indicates that melatonin originating from the pineal gland and melatonin synthesized by brain tissue both contribute to the high level of melatonin in CSF. The major source of melatonin in CSF seems to come from the direct release of melatonin from the pineal gland into the pineal recess of the third ventricle. This leads to a melatonin concentration gradient in CSF as the fluid flows through the ventricular system including the aqueduct, fourth ventricle, and subarachnoid space. Melatonin in the CSF is speculated to protect the surrounding brain structures from oxidative and nitrosative stress. A low level of melatonin in CSF may relate to the etiology of neurodegenerative diseases which have elevated oxidative stress, e.g., Alzheimer disease. Decreased melatonin levels in CSF have been observed in patients with this neurodegenerative condition. Long term melatonin supplementation may retard the progress of some neurodegenerative diseases. This conclusion is based on a variety of animal studies and several small scale clinical investigations.

## Figures and Tables

**Table1:** Summary of the Presumed Physiological Concentrations of Melatonin in Human CSF

Reference	Year						Melatonin (pg/ml)
		Collection Time	Collection Site	Ages (Yr)	Method	Melatonin Form	
Rousseau *et al.* [54]	1999	08:00-09:00h	Lumbar cistern	25.3 ± 4.5	RIA	Free	32.5 ± 25.5
Rousseau *et al.* [54]	1999	08:00-09:00h	Lumbar cistern	25.3 ± 4.5	RIA	Free	32.5 ± 25.5
Rousseau *et al.* [54]	1999	08:00-09:00h	Lumbar cistern	25.3 ± 4.5	RIA	Free	32.5 ± 25.5
Liu *et al.*[31]	1999	1-12 h after death	Ventricular	76 ± 1.4	RIA	Free	273 ± 47
Rizzo *et al.* [53]	2002	Night	Lumbar cistern	N/A	HPLC	Free + bound	28.6 ± 7.0
Rizzo *et al.* [53]	2002	Night	Lumbar cistern	N/A	HPLC	Free + bound	28.6 ± 7.0
Zhou *et al.* [72]	2003	1-12 h after death	Ventricule	76 ± 2	RIA	Free	280 ± 64
Longatti *et al.*[34]	2004	Day time	Third ventricule	N/A	N/A	Free	542
Longatti *et al.* [35]	2007	Day time	Third ventricule	60.3 ± 17.9	HPLC	Free + bound	442 ± 45
Seifman *et al.* [34]	2008	09:00h	ventricule	30-74	ELISA	Free	1.47 ± 0.35
Leston *et al.*[30]	2010	08:10-11:10 h	Third ventricule	26-68	RIA	Free	8.69 ± 2.75
